# Sociodemographic, Self-Efficacy, and Health-Related Factors According to the Stages of Change in Physical Activity and Dietary Management Among Patients with Metabolic Syndrome: A Secondary Analysis of a Cross-Sectional Study

**DOI:** 10.3390/nursrep16100347

**Published:** 2026-09-23

**Authors:** Haejung Lee, DaeEun Lee

**Affiliations:** 1 College of Nursing, Pusan National University, Yangsan 50612, Republic of Korea; haejung@pusan.ac.kr; 2 Research Institute of Nursing Science, Pusan National University, Yangsan 50612, Republic of Korea

**Keywords:** diet, exercise, health behavior, metabolic syndrome, transtheoretical model

## Abstract

**Purpose**: This study aimed to examine differences in sociodemographic characteristics, self-efficacy, and health-related factors across the stages of change for physical activity and dietary management among patients with metabolic syndrome (MetS). **Methods**: This secondary analysis included 208 adults aged 40–<70 with MetS who were recruited from a university hospital in South Korea. Participants had either a history of cardiovascular disease (CVD) or an intermediate-to-high 10-year CVD risk. Data were analyzed using descriptive statistics, chi-square test or Fisher’s exact test, Mann–Whitney U test, and Kruskal–Wallis test. Statistical analyses were performed using SPSS version 22.0 and RStudio version 2026.08.1. **Results**: Across the stages of change for physical activity, significant differences were observed in age, healthy diet, self-care, and current smoking status. Across the stages of change for dietary management, significant differences were observed in healthy diet and self-care. When participants were classified into no-action, single-action, and double-action groups based on their stages of change for physical activity and dietary management, significant differences were found in age, employment status, healthy diet, self-care, and current smoking status. **Conclusions**: These findings suggest that interventions for patients with MetS should be tailored to their stages of change and health-related characteristics. In particular, interventions that address both physical activity and dietary management and strengthen collaborative relationships with healthcare providers may help promote health behavior change in this population.

## 1. Introduction

Metabolic syndrome (MetS) is a common lifestyle-related condition characterized by the presence of three or more metabolic abnormalities, including abdominal obesity, elevated blood pressure, hyperglycemia, hypertriglyceridemia, and low high-density lipoprotein (HDL) cholesterol. Globally, the prevalence of MetS increased from 11.9% in 2000 to 28.4% in 2023, rising from 14.7% to 31.0% among women and from 9.0% to 25.7% among men [1]. In South Korea, the prevalence increased from 22.8% in 2007 to 28.6% in 2022, with a marked increase among men from 24.5% to 36.8%, while it slightly decreased among women from 20.6% to 19.5% [2]. The Korean MetS Fact Sheet [3] also reported a high prevalence of individual MetS components, including abdominal obesity (40.0%), hyperglycemia (33.3%), hypertension (29.3%), hypertriglyceridemia (25.3%), and low HDL cholesterol (25.6%).

MetS substantially increases the risk of adverse health outcomes. Compared with those without MetS, individuals with MetS have a multiadjusted hazard ratio (HR) of approximately 1.24 (95% CI [confidence interval] 1.16–1.33) for all-cause mortality and an approximately 5.15-fold higher risk of diabetes mellitus mortality (95% CI 3.15–8.43) [4]. MetS is also associated with increased risks of cardiovascular and cerebrovascular diseases and myocardial infarction [5]. Because MetS is closely related to modifiable lifestyle behaviors, improving physical activity and dietary habits is an important component of its prevention and management.

Physical activity and dietary management are key lifestyle strategies for reducing cardiometabolic risk among individuals with MetS [6,7]. Previous studies have shown that these behaviors are associated with lower cardiovascular and all-cause mortality [8] and that combining dietary management with physical activity may provide greater improvements in fasting blood glucose, waist circumference, body weight, and body mass index than dietary management alone [9]. However, initiating and maintaining healthy lifestyle behaviors can be challenging. For example, only 27.6% of individuals at risk for MetS have been reported to meet recommended physical activity levels [10]. These findings underscore the need for behavioral interventions that account for individuals’ readiness to change.

The Transtheoretical Model (TTM) provides a useful framework for understanding and promoting health behavior change. The TTM conceptualizes behavior change as a progression through stages, from precontemplation and contemplation to preparation, action, and maintenance [11]. Individualized interventions tailored to the stages of change in physical activity and diet based on the TTM have been shown to improve healthy lifestyle behaviors among patients with MetS [12]. Because the stages of change may vary across health behaviors among patients with MetS, assessing these stages may help inform individualized strategies for promoting healthy behaviors. Moreover, different health behaviors may be at different stages of change within the same individual. For example, Holmen et al. [13] reported that 42.0% of participants were in the action stage for physical activity, compared with only 21.0% for dietary management. Furthermore, MetS is influenced by multiple health behaviors, including physical inactivity, unhealthy diet, smoking, and alcohol consumption [6,7,9]. Therefore, examining the stages of change for physical activity and dietary management both separately and in combination may provide a more comprehensive understanding of behavioral readiness among patients with MetS [14].

Accordingly, this study aimed to examine differences in sociodemographic characteristics, self-efficacy, and health-related factors according to the individual and combined stages of change for physical activity and dietary management among Korean adults with MetS. The findings may provide foundational evidence for developing stage-tailored interventions that address multiple health behaviors in this population.

## 2. Methods

### 2.1. Study Design

This cross-sectional study used data derived from a case-control study entitled “Comparison of Health Indicators According to the Presence or Absence of Acute Coronary Syndrome (ACS) in Patients with Metabolic Syndrome.” The present study aimed to classify patients with MetS according to their stages of change in physical activity and dietary management and to compare sociodemographic characteristics, self-efficacy, and health-related factors across individual and combined stages of change in these two health behaviors.

### 2.2. Participants

Participants were outpatients aged 40 to under 70 years with MetS who were receiving treatment at the Department of Endocrinology or the Cardiovascular Center of Pusan National University Yangsan Hospital. In the parent study, eligible patients were classified into a case group (history of percutaneous coronary intervention or coronary artery bypass grafting for acute coronary syndrome [ACS]) or a control group (no history of ACS but an intermediate-to-high 10-year cardiovascular disease [CVD] risk based on the Predicting Risk of Cardiovascular Disease [PREVENT] score [≥7.5%]) [15]. Exclusion criteria were participation in other lifestyle interventions, clinical instability, and communication impairment.

MetS was defined by the presence of at least three of the following five criteria [16]: (a) abdominal obesity (waist circumference (WC) ≥ 90 cm for men, ≥85 cm for women, or BMI ≥ 25.0 kg/m^2^ when WC was unavailable) [17]; (b) blood pressure ≥ 130/85 mmHg or use of antihypertensive medication; (c) fasting blood glucose ≥ 100 mg/dL or use of glucose-lowering medications; (d) low HDL cholesterol (<40 mg/dL for men, <50 mg/dL for women, or use of lipid-lowering medication); and (e) triglycerides ≥ 150 mg/dL or treatment for hypertriglyceridemia.

The present study analyzed the full sample of 208 participants because the core study variables had no missing values. Missing data were present for some clinical indicators (Table 1). To assess potential bias due to missing data, a sensitivity analysis was conducted using multiple imputation by chained equations (MICE; *N* = 208) [18], which yielded results consistent with those from the original dataset (Table S1).

The dataset used in this study was previously analyzed by Lee and Lee [19] to examine associations between dietary patterns and clinical indicators. That study included 185 participants after excluding those with missing dietary intake (*n* = 21) or clinical indicator data (*n* = 2), and assessed dietary patterns based on 24-h dietary recalls. In contrast, the present study assessed healthy dietary behavior using the Mini Dietary Assessment and examined stages of change in physical activity and dietary management. Although the two studies used the same underlying dataset, they addressed distinct research questions and differed in dietary measures, outcomes, and analytical approaches.

The required sample size was estimated using G*Power version 3.1.9.7. Because the primary analysis involved comparisons among three groups, a one-way analysis of variance (ANOVA) was used for the a priori sample size calculation as a conservative approximation for the Kruskal–Wallis test. Assuming a medium effect size (f = 0.25), a significance level of α = 0.05, and 90% power, a minimum total sample size of 206 participants was required for three groups. The available sample of 208 participants therefore met the estimated sample size requirement for the primary group comparison.

### 2.3. Measures

#### 2.3.1. Sociodemographic and Clinical Characteristics

The sociodemographic characteristics included age, sex, educational level, marital status, employment status, and monthly income. Age was categorized into 40–49 years, 50–59 years, and 60–<70 years. Educational level was categorized as ≤high school graduation, and ≥university graduation. Monthly income was divided into <2 million KRW, 2 million to 4.9 million KRW, and ≥5 million KRW. Clinical characteristics included HbA1c, fasting glucose, WC, systolic blood pressure (SBP), diastolic blood pressure (DBP), and lipid profile, including total cholesterol, HDL-cholesterol, low-density lipoprotein (LDL)-cholesterol, and triglycerides. A history of acute coronary syndrome (ACS) underwent percutaneous coronary intervention or coronary artery bypass grafting was also assessed.

#### 2.3.2. The Stages of Change for Physical Activity and Dietary Management

The stages of change for health behavior [11] were assessed using a five-stage classification based on the Korean version of the instrument, which was approved by the original translator [20]. Participants were asked to select the single option that best represented their current stage of change for physical activity and dietary management. The stages were defined as follows: Physical activity: (a) Precontemplation: had no intention to exercise; (b) Contemplation: recognized the need to exercise, but was not yet engaging in regular exercise; (c) Preparation: was interested in exercising and making specific preparations, such as seeking information about exercise facilities; (d) Action: had been exercising for less than 6 months; and (e) Maintenance: had been exercising continuously for 6 months or longer. Dietary management: (a) Precontemplation: had no intention to manage their diet; (b) Contemplation: recognized the need for dietary management but was not yet engaging in dietary management; (c) Preparation: was interested in dietary management and making specific preparations, such as seeking dietary information; (d) Action: had been managing their diet for less than 6 months; and (e) Maintenance: had been managing their diet continuously for 6 months or longer.

Based on the literature [11], the five stages of change were reclassified into two groups: a preparation group (precontemplation, contemplation, and preparation) and an action group (action and maintenance). Given that the absence of even a single healthy behavior may substantially influence disease outcomes [14], participants were then classified into three groups according to the combined stages of change in physical activity and dietary management: a no-action group (preparation group for both behaviors), a single-action group (action group for one behavior and preparation group for the other), and a double-action group (action group for both behaviors).

#### 2.3.3. Self-Efficacy

Self-efficacy was measured using the Korean version of the Self-Efficacy for Managing Chronic Disease 6-Item Scale (SECD-6-K) [21], which was translated from the original SECD-6 developed by Lorig et al. [22] to evaluate the self-efficacy of patients with chronic diseases in managing their health problems. The SECD-6-K consists of 6 items across two subscales: symptom management and health behavior. Each item is rated on a 10-point Likert scale ranging from 1 (“not at all confident”) to 10 (“totally confident”), with higher scores indicating a higher level of self-efficacy for chronic disease management. The Cronbach’s α was 0.91 for the original scale [22], 0.96 for the Korean version [21], and 0.93 in this study.

#### 2.3.4. Health-Related Factor

##### Healthy Diet

Healthy diet was assessed using the Mini Dietary Assessment (MDA) [23] and the dietary recall method derived from the Korean National Health and Nutrition Examination Survey (2007). The MDA includes items evaluating the intake levels of major food groups (such as milk and dairy products, meat, fish, eggs, legumes, tofu, vegetables, and fruits), items encouraging the reduction of excessive intake (e.g., simple sugars from fried or stir-fried foods and ice cream), and items regarding a balanced diet (e.g., regular meals and diversity in food consumption). This instrument consists of 10 items, each rated on a 3-point scale: “strongly agree,” “agree,” and “strongly disagree.” Scores of 5, 3, and 1 point are assigned to these responses, respectively, based on the desirability of the dietary practice. The total score ranges from 10 to 50, with higher scores indicating a healthier diet. To evaluate the criterion validity of this instrument, a comparative analysis of the Healthy Eating Index (HEI) and the MDA was conducted among adults in their 30s and 40s, which demonstrated no significant difference between the two instruments [23]. In this study, Cronbach’s α was 0.65.

##### Self-Care

Self-care was assessed using the Korean version of the Partners in Health Scale (PIH-K) [24], which was translated from the original PIH scale developed to measure the self-care of patients with chronic diseases [25]. The PIH-K consists of 12 items across four subscales: coping (4 items), partnership in treatment (4 items), recognition and managing symptoms (2 items), and knowledge (2 items). Each item is rated on a 9-point Likert scale ranging from 0 (“not at all”) to 8 (“very much”). The total score ranges from 0 to 96, with higher scores indicating a higher level of self-care. The Cronbach’s α for both the original [25] and Korean [24] versions was 0.86 and 0.91 in the present study.

##### Sleep Quality

Sleep quality was assessed using the Korean version of the Pittsburgh Sleep Quality Index (PSQI-K) [26], which was translated from the original PSQI [27]. The instrument is a self-report questionnaire designed to evaluate sleep quality and disturbances over the past month. It consists of 19 items across seven subscales: subjective sleep quality, sleep latency, sleep duration, habitual sleep efficiency, sleep disturbances, use of sleeping medication, and daytime dysfunction. Each item is rated from 0 to 3, with the total score ranging from 0 to 21; higher scores indicate poorer sleep quality. With a cut-off score of 5 points to distinguish between adults with and without sleep problems, the instrument demonstrated a sensitivity of 0.72 and a specificity of 0.88 [28]; thus, this classification criterion was also adopted in the present study. Cronbach’s α was 0.83 for the original scale [27] and 0.84 for the PSQI-K [26], compared to 0.69 in the present study.

##### Current Drinker and Smoker

Drinking and smoking statuses were assessed using the questionnaire designed for this study. Regarding drinking status, participants who selected “did not drink at all in the past month” to the item “How often have you consumed alcohol within the past month?” were classified into the non-drinker group. Those who selected any of the remaining responses (“once a month,” “2–4 times a month,” “2–3 times a week,” or “4 or more times a week”) were classified into the current drinker group. Regarding smoking status, participants who responded “yes” to the item “Have you smoked within the past month?” were classified into the current smoker group, while those who responded with the remaining options (“no” or “used to smoke but quit”) were classified into the non-smoker group.

### 2.4. Data Collection Procedure

Data collection for the primary study was conducted from April 2024 to August 2024, with five research assistants participating in the process. To identify potentially eligible participants, electronic medical records were reviewed on the day prior to recruitment to verify the diagnosis of MetS. When potential participants visited the clinic for their outpatient appointments, the purpose and procedures of the study were explained to them. Data collection was then performed for those who provided voluntary informed consent. The survey took approximately 20–25 min and was conducted in an empty outpatient examination room, followed by anthropometric measurements.

### 2.5. Data Analysis

Statistical analysis was performed using RStudio version 2026.08.1 (Posit Software, PBC, Boston, MA, USA) and SPSS version 22.0 (IBM Corp., Armonk, NY, USA), with two-tailed tests at a significance level of *p* < 0.05. For sensitivity analysis, missing data imputation was conducted using the ‘mice’ package version 3.19.0 in RStudio [18].

Descriptive statistics, including means, standard deviations, frequencies, and percentages, were calculated to summarize participant characteristics. Normality was assessed using the Kolmogorov–Smirnov test. Due to the non-normal distribution of continuous variables, group differences were assessed using the Mann–Whitney U test for two groups and the Kruskal–Wallis test for three groups. When the Kruskal–Wallis test was significant, Dunn’s post hoc test with Bonferroni adjustment was performed for pairwise comparisons. To account for multiple testing across outcomes, *p*-values from the Kruskal–Wallis tests were adjusted using the Benjamini–Hochberg false discovery rate (FDR) procedure.

Effect sizes and 95% confidence intervals (CIs) for continuous variables were calculated using the rank-biserial correlation for two groups and rank epsilon squared for three or more groups. For categorical variables, effect sizes were measured using Cramér’s V and the 95% CIs were obtained by inverting the noncentral chi-square distribution.

### 2.6. Ethical Consideration

This study is a secondary data analysis approved as exempt from review by the Institutional Review Board (IRB) of Pusan National University (IRB No. 2025-10-088, approved on 17 December 2025).

## 3. Results

### 3.1. Sociodemographic Characteristics of the Participants

The sociodemographic characteristics of the 208 participants are summarized in Table 1. The mean age was 60.68 ± 6.25 years, and 136 participants (65.4%) were aged 60 years or older. The majority of participants were male (*n* = 165, 79.3%). A total of 120 participants (57.7%) had a high school education or lower, 161 (77.4%) were married, 137 (65.9%) were employed, and 51 (24.5%) had a monthly income of <2 million KRW.

Regarding clinical characteristics, 127 participants (61.1%) had a BMI ≥ 25 kg/m^2^. The mean HbA1c and glucose levels were 6.73 ± 0.93% and 143.02 ± 54.20 mg/dL, respectively. The mean SBP and DBP were 124.57 ± 15.23 and 74.61 ± 11.64 mmHg, respectively. The mean LDL-C, total cholesterol, and triglyceride levels were 60.05 ± 28.48, 141.46 ± 32.54, and 163.25 ± 85.56 mg/dL, respectively. The mean HDL-C levels were 47.72 ± 9.82 mg/dL in males and 52.62 ± 11.19 mg/dL in females. A total of 104 participants (50.0%) had a history of ACS.

The mean scores for self-efficacy, healthy diet, and self-care were 6.64 ± 2.06, 32.53 ± 6.04, and 71.10 ± 13.96, respectively. A total of 126 participants (60.6%) had poor sleep quality (score ≥ 5). In addition, 101 participants (48.6%) were current drinkers and 56 (26.9%) were current smokers. Regarding the stages of change, 109 participants (52.4%) were classified into the action group for physical activity, and 79 (38.0%) were classified into the action group for dietary management. Based on the combined stages of change for both behaviors, 76 participants (36.5%) were classified into the no-action group, 76 (36.5%) into the single-action group, and 56 (27.0%) into the double-action group.

### 3.2. Differences in Sociodemographic Characteristics, Self-Efficacy, and Health-Related Factors According to Stages of Change in Physical Activity and Dietary Management

The characteristics of participants according to their stages of change in physical activity and dietary management are presented in Table 2. For physical activity, significant differences were observed in age, healthy diet, self-care, and smoking status between the preparation and action groups (adjusted *p* < 0.05). Participants in the action group were older than those in the preparation group (61.63 ± 6.32 vs. 59.64 ± 6.04 years) and had higher healthy diet scores (33.83 ± 5.88 vs. 31.09 ± 5.92) and self-care scores (74.41 ± 13.64 vs. 67.44 ± 13.45). The proportion of nonsmokers was higher in the action group than in the preparation group (81.7% vs. 63.6%).

For dietary management, significant differences were observed in healthy diet and self-care scores between the preparation and action groups (adjusted *p* < 0.05). Participants in the action group had higher healthy diet scores than those in the preparation group (34.58 ± 5.85 vs. 31.27 ± 5.84) and higher self-care scores (76.95 ± 12.18 vs. 67.51 ± 13.81).

### 3.3. Differences in Sociodemographic Characteristics, Self-Efficacy, and Health-Related Factors According to the Combined Stages of Change

The characteristics of participants according to the combined stages of change are presented in Table 3. Significant differences were observed in age, employment status, healthy diet, self-care, and current smoking status across the three groups. Participants in the double-action group (62.54 ± 6.42 years) were older than those in the no-action (59.57 ± 6.07 years) and single-action group (60.43 ± 6.09 years). The proportion of employed participants was 73.7% (*n* = 56) in the single-action group. Healthy diet scores were significantly lower in the no-action group than in the single-action and double-action groups (30.13 ± 5.70 vs. 33.32 ± 5.67 and 34.71 ± 5.97, respectively). Similarly, self-care scores were lower in the no-action group than in the single-action and double-action groups (65.11 ± 13.24 vs. 72.24 ± 13.30 and 77.68 ± 12.56, respectively). The proportion of nonsmokers was lower in the no-action group than in the double-action group (64.5% vs. 89.3%).

## 4. Discussion

This study aimed to examine differences in sociodemographic characteristics, self-efficacy, and health-related factors according to the stages of change for physical activities and dietary management among patients with MetS. Significant differences were observed in age, healthy diet, self-care, and smoking status according to the stages of change for physical activity, and in healthy diet and self-care according to the stages of change for dietary management. When the two behaviors were considered jointly, significant differences were found in age, employment status, healthy diet, self-care, and smoking status across the no-action, single-action, and double-action groups. These findings suggest that readiness to engage in health behaviors may vary according to individual characteristics and that physical activity and dietary management may progress at different rates. Although the cross-sectional design precludes causal inference, identifying characteristics associated with different stages of change may help inform more individualized behavioral interventions for patients with MetS.

An important finding was that physical activity and dietary management showed different distributions across the stages of change. More than half of the participants (52.4%, *n* = 109) were in the action group for physical activity, whereas only 38.0% (*n* = 79) were in the action group for dietary management. In addition, only 27.0% (*n* = 56) were classified in the double-action group, indicating that simultaneous engagement in both behaviors was less common than engagement in either behavior alone. This finding suggests that physical activity and dietary management may not necessarily progress concurrently. Physical activity may be more directly controlled by individual decisions and opportunities for activity, whereas dietary behaviors may be influenced more strongly by food availability, social circumstances, and other environmental factors. Although these factors were not directly assessed in this study, the different distributions across behavioral stages support the need to assess readiness for physical activity and dietary management separately rather than assuming that progress in one behavior reflects progress in the other. For patients who are ready to change one behavior but not the other, interventions could prioritize the behavior with greater readiness while gradually addressing the second behavior.

Self-care was a consistent factor associated with behavioral stage. Participants in the action group for physical activity and dietary management had higher self-care scores than those in the preparation groups. Similarly, participants in the single-action and double-action groups had higher self-care scores than those in the no-action group. The consistency of this finding across individual and combined behavioral classifications suggests that self-care may be an important characteristic of patients who are more actively engaged in health behaviors. Self-care encompasses behaviors through which individuals monitor and manage their health and may therefore be relevant to the adoption of multiple health-promoting behaviors. However, because the present study was cross-sectional, it cannot be determined whether greater self-care facilitates progression to more advanced stages or whether engagement in health behaviors is accompanied by greater self-care. Nevertheless, assessing self-care may be useful when identifying patients who may require additional support for initiating or maintaining lifestyle changes.

In this study, age differed according to the stages of change for both individual and combined health behaviors. Participants in the physical activity Action group were older than those in the Preparation group, and the double-action group was older than the no-action group. This pattern suggests that older participants may have been more likely to engage in or maintain health-promoting behaviors. More than half of the participants were aged 60–<70 years, an age group in which MetS is relatively common in Korea [3]. Previous studies have also reported that several components of MetS, including elevated blood glucose and SBP and lower high-density lipoprotein cholesterol levels, are associated with older age [29]. However, the mechanisms underlying the observed association between age and behavioral stage cannot be determined from the present data. Differences in occupational demands, social activities, or available time may contribute to differences in readiness for health behavior change, particularly among middle-aged adults. Therefore, interventions may benefit from considering age-related lifestyle demands when supporting physical activity and dietary management.

Smoking status differed according to the stages of change for physical activity and the combined behavioral stages. The proportion of nonsmokers was higher among participants in more advanced behavioral groups, particularly in the double-action group. This pattern may indicate that health behaviors tend to cluster rather than occur independently [14]. Patients who engage in one health-promoting behavior may also be more likely to engage in other behaviors [30], although the cross-sectional nature of the study prevents conclusions about the direction of these relationships. This finding supports the value of assessing multiple health behaviors [14,30] when providing lifestyle management for patients with MetS. Rather than addressing physical activity or dietary management in isolation, healthcare providers may consider smoking status and other health behaviors when developing individualized intervention plans.

Employment status was associated with the combined stages of change. Employment was more common in the no-action and single-action groups than in the double-action group. Previous research [31] has indicated that health behaviors among individuals with MetS may vary according to occupational characteristics, and qualitative studies have identified long working hours and financial burdens related to medical care as potential barriers to self-management. In addition, office workers have been reported to spend more time in sedentary behavior and less time in light and moderate-to-vigorous physical activity than individuals in other occupational groups [32]. These findings suggest that occupational circumstances may influence the feasibility of maintaining multiple health behaviors simultaneously. However, the present study did not assess job type, working hours, shift work, or other specific occupational characteristics. Therefore, future research should examine these factors to clarify how workplace environments and occupational demands may affect readiness for concurrent physical activity and dietary management.

In contrast, self-efficacy did not differ significantly according to the stages of change. This finding differs from previous research [33] showing that self-efficacy enhancement programs were associated with improvements in metabolic risk factors, including waist circumference, SBP, DBP, and high-density lipoprotein cholesterol. One possible explanation is that the self-efficacy measure used in the present study assessed general self-efficacy for chronic disease management rather than confidence in performing specific health behaviors. General self-efficacy may therefore not adequately capture the behavioral confidence required to initiate or maintain physical activity and dietary changes. Future studies should consider using behavior-specific self-efficacy measures for physical activity and dietary management to determine whether confidence in specific behaviors is more closely associated with stages of change and to identify appropriate targets for intervention.

Among the participants with MetS, 79.3% were male. This sex distribution may reflect sex-specific differences in the prevalence and behavioral risk factors of MetS in South Korea [34,35]. National data have shown a higher prevalence of MetS among Korean men than women, with men also reporting higher levels of smoking and alcohol consumption [34,35]. In contrast, the risk of MetS increases among women after menopause, particularly in association with abdominal obesity and changes in body fat distribution [2]. In addition, dietary patterns characterized by high sodium intake have been associated with MetS risk in Korea [36]. These findings highlight the importance of considering sex-specific and culturally relevant dietary and lifestyle characteristics when developing interventions for patients with MetS in the Korean healthcare context.

In this study, 60.6% of participants with MetS experienced poor sleep quality. Previous studies have reported associations between poor sleep quality, short sleep duration, prolonged sleep latency, and increased metabolic and cardiovascular risks among individuals with MetS [37,38]. These findings suggest that sleep may be an important health behavior to consider alongside physical activity and dietary management. Therefore, individualized assessment of sleep characteristics may also be considered when developing comprehensive lifestyle interventions for patients with MetS.

This study has several limitations. First, as a secondary analysis employing a cross-sectional design, causal relationships cannot be established. Longitudinal studies with larger and more diverse samples are needed to examine whether the characteristics identified in this study predict progression across stages of change. Second, lifestyle-related variables, including sleep quality, dietary behaviors, self-care, and stages of change, were assessed using self-reported questionnaires and may therefore be subject to response bias. Future studies could incorporate objective measures where appropriate, particularly for physical activity and other behaviors for which objective assessment is feasible. Third, the five stages of change were collapsed into preparation and action groups for the individual behavior analyses. Although this approach facilitated interpretation, it may have obscured meaningful differences among the five original stages. Future research should examine all five stages when sample sizes permit. Fourth, participants were recruited from a single university hospital, which may limit the generalizability of the findings to other clinical settings and populations. Finally, the study involved multiple statistical comparisons, increasing the potential for type I error. Although statistical correction was applied, findings with borderline statistical significance should be interpreted cautiously and considered exploratory.

Despite these limitations, this study provides evidence that patients with MetS differ in their readiness for physical activity and dietary management according to sociodemographic and health-related characteristics. The findings suggest that readiness for physical activity and dietary management should be assessed separately and that self-care may be a useful characteristic to consider when identifying patients who are more or less ready for behavior change. The relatively small proportion of participants in the double-action group (27.0%) further suggests that concurrent engagement in both behaviors may be more challenging than changing either behavior alone. From a clinical perspective, nurses and other healthcare providers may use stage assessment to identify patients’ current readiness and prioritize the behavior for which they are most amenable to change. Based on the assessment, stage-tailored individualized support could progress toward additional health behaviors support rather than applying the same lifestyle intervention to all patients. Attention to sociodemographic characteristics, such as employment, age, and smoking status, may further help tailor the intensity and content of behavioral support.

## 5. Conclusions

This study identified differences in sociodemographic and health-related characteristics according to the stages of change for physical activity and dietary management among patients with MetS. Physical activity and dietary management showed different patterns of behavioral readiness, with a lower proportion of participants reaching the action stage for dietary management. More advanced stages of change were characterized by higher levels of self-care, while employment and educational characteristics differed across the combined behavioral stages. These findings suggest that health behavior change among patients with MetS may vary according to individual characteristics and across different health behaviors. Therefore, interventions should be tailored to patients’ stages of change and individual characteristics, with separate assessment of readiness for physical activity and dietary management. Incorporating self-care support, structured dietary management, and collaborative patient–provider relationships may help facilitate health behavior change in this population.

## Figures and Tables

**Table 1:** Sociodemographic characteristics of the participants (*N* = 208).

Characteristics	Categories	*n* (%) or M ± SD
Age (years)		60.68 ± 6.25
	40–49	17 (8.2)
	50–59	55 (26.4)
	60–<70	136 (65.4)
Sex	Male	165 (79.3)
	Female	43 (20.7)
Level of education	≤High school	120 (57.7)
	≥University	88 (42.3)
Marital status	Married	161 (77.4)
	Single	47 (22.6)
Employment status	Employed	137 (65.9)
	Unemployed	71 (34.1)
Monthly income (million won)	<2	51 (24.5)
	2–4.9	89 (42.8)
	≥5	68 (32.7)
Body mass index (kg/m^2^)		26.14 ± 3.50
	<18.5	2 (1.0)
	18.5–22.9	34 (16.3)
	23.0–24.9	45 (21.6)
	≥25.0	127 (61.1)
HbA1c (%) (*n* = 205)		6.73 ± 0.93
Glucose (mg/dL) (*n* = 203)		143.02 ± 54.20
Waist circumference (cm) (*n* = 207)		92.62 ± 8.93
	Male (*n* = 164)	93.58 ± 8.45
	Female (*n* = 43)	88.96 ± 9.85
Systolic blood pressure (mmHg)		124.57 ± 15.23
Diastolic blood pressure (mmHg)		74.61 ± 11.64
**Lipid profiles** (mg/dL) (*n* = 204)		
High-density lipoprotein cholesterol		48.73 ± 10.28
	Male (*n* = 162)	47.72 ± 9.82
	Female (*n* = 42)	52.62 ± 11.19
Low-density lipoprotein cholesterol		60.05 ± 28.48
Total cholesterol		141.46 ± 32.54
Triglyceride		163.25 ± 85.56
Cardiovascular disease	Yes	104 (50.0)
	No	104 (50.0)
Self-efficacy		6.64 ± 2.06
**Health-related factor**		
Healthy diet		32.53 ± 6.04
Self-care		71.10 ± 13.96
Sleep quality		7.17 ± 3.66
	<5	82 (39.4)
	≥5	126 (60.6)
Current drinker	Yes	101 (48.6)
	No	107 (51.4)
Current smoker	Yes	56 (26.9)
	No	152 (73.1)
**Stages of behavioral change ^a^**		
Physical activity	Preparation group	99 (47.6)
	Precontemplation	8 (3.8)
	Contemplation	57 (27.4)
	Preparation	34 (16.3)
	Action group	109 (52.4)
	Action	24 (11.5)
	Maintenance	85 (40.9)
Dietary management	Preparation group	129 (62.0)
	Precontemplation	16 (7.7)
	Contemplation	74 (35.6)
	Preparation	39 (18.8)
	Action group	79 (38.0)
	Action	21 (10.1)
	Maintenance	58 (27.9)
**Combined stages of behavioral change ^b^**	No-action group	76 (36.5)
	Single-action group	76 (36.5)
	Double-action group	56 (27.0)

Note. M = Mean; SD = Standard deviation. **^a^** Preparation group = precontemplation, contemplation, and preparation stages; action group = action and maintenance stages. **^b^** No-action group = preparation group for both physical activity and dietary management; single-action group = reclassified action group for either physical activity or dietary management; double-action group = reclassified action group for both physical activity and dietary management.

**Table 2:** Differences in sociodemographic characteristics, self-efficacy, and health-related factors by stages of change in physical activity and diet (*N* = 208).

Variables	Physical Activity		x^2^ or Z ^a^	(BH-Adjusted *p*) ^b^	Effect Size ^c^	(95% CI) ^d^	Dietary Management		x^2^ or Z ^a^	(BH-Adjusted *p*) ^b^	Effect Size ^c^	(95% CI) ^d^
	Preparation G (*n* = 99)	Action G (*n* = 109)					Preparation G (*n* = 129)	Action G (*n* = 79)				
	*n* (%) or M ± SD						*n* (%) or M ± SD					
Age (years)	59.64 ± 6.04	61.63 ± 6.32	−2.91	(0.012)	−0.23	(−0.38, −0.08)	60.02 ± 6.09	61.76 ± 6.40	−2.41	(0.069)	−0.20	(−0.35, −0.04)
40–49	8 (8.1)	9 (8.3)	7.92	(0.018) ^e^			11 (8.5)	6 (7.6)	2.79	(0.265) ^e^		
50–59	35 (35.4)	20 (18.3)					39 (30.2)	16 (20.3)				
60–<70	56 (56.6)	80 (73.4)					79 (61.2)	57 (72.2)				
Sex												
Male	82 (82.8)	83 (76.1)	1.41	(0.339)	0.08	(0.00, 0.22)	106 (82.2)	59 (74.7)	1.68	(0.345)	0.09	(0.00, 0.23)
Female	17 (17.2)	26 (23.9)					23 (17.8)	20 (25.3)				
Level of education												
≤High school	59 (59.6)	61 (56.0)	0.28	(0.646)	0.04	(0.00, 0.17)	72 (55.8)	48 (60.8)	0.49	(0.571)	0.05	(0.00, 0.18)
≥University	40 (40.4)	48 (44.0)					57 (44.2)	31 (39.2)				
Marital status												
Married	71 (71.7)	90 (82.6)	3.49	(0.134)	0.13	(0.00, 0.27)	99 (76.7)	62 (78.5)	0.09	(0.771)	0.02	(0.00, 0.15)
Single	28 (28.3)	19 (17.4)					30 (23.3)	17 (21.5)				
Employment status												
Employed	71 (71.7)	66 (60.6)	2.88	(0.167)	0.12	(0.00, 0.25)	91 (70.5)	46 (58.2)	3.31	(0.180)	0.13	(0.00, 0.26)
Unemployed	28 (28.3)	43 (39.4)					38 (29.5)	33 (41.8)				
Monthly income (million won)												
<2	24 (24.2)	27 (24.8)	2.10	(0.414)	0.10	(0.00, 0.22)	28 (21.7)	23 (29.1)	1.63	(0.571)	0.09	(0.00, 0.21)
2–4.9	47 (47.5)	42 (38.5)					56 (43.4)	33 (41.8)				
≥5	28 (28.3)	40 (36.7)					45 (34.9)	23 (29.1)				
Cardiovascular disease												
Yes	51 (51.5)	53 (48.6)	0.17	(0.677)	0.03	(0.00, 0.16)	70 (54.3)	34 (43.0)	2.47	(0.251)	0.11	(0.00, 0.25)
No	48 (48.5)	56 (51.4)					59 (45.7)	45 (57.0)				
Self-efficacy	6.43 ± 1.98	6.84 ± 2.11	−1.87	(0.134)	−0.15	(−0.30, 0.01)	6.61 ± 1.97	6.70 ± 2.21	−0.95	(0.492)	−0.08	(−0.24, 0.08)
**Health-related factor**												
Healthy diet	31.09 ± 5.92	33.83 ± 5.88	−3.11	(0.012)	−0.25	(−0.39, −0.10)	31.27 ± 5.84	34.58 ± 5.85	−3.37	(0.005)	−0.28	(−0.42, −0.12)
Self-care	67.44 ± 13.45	74.41 ± 13.64	−3.87	(0.001)	−0.31	(−0.45, −0.16)	67.51 ± 13.81	76.95 ± 12.18	−5.28	(<0.001)	−0.44	(−0.56, −0.30)
Sleep quality	6.80 ± 3.55	7.51 ± 3.74	−1.47	(0.142) ^e^			7.22 ± 3.72	7.10 ± 3.59	0.18	(0.856) ^e^		
<5	44 (44.4)	38 (34.9)	2.00	(0.257)	0.10	(0.00, 0.23)	52 (40.3)	30 (38.0)	0.11	(0.771)	0.02	(0.00, 0.15)
≥5	55 (55.6)	71 (65.1)					77 (59.7)	49 (62.0)				
Current drinker												
Yes	52 (52.5)	49 (45.0)	1.19	(0.358)	0.08	(0.00, 0.21)	67 (51.9)	34 (43.0)	1.55	(0.345)	0.09	(0.0, 0.22)
No	47 (47.5)	60 (55.0)					62 (48.1)	45 (57.0)				
Current smoker												
Yes	36 (36.4)	20 (18.3)	8.56	(0.012)	0.20	(0.07, 0.34)	41 (31.8)	15 (19.0)	4.08	(0.141)	0.14	(0.00, 0.28)
No	63 (63.6)	89 (81.7)					88 (68.2)	64 (81.0)				

Note. CI = Confidence intervals; G = group; M = Mean; SD = Standard deviation. Preparation = precontemplation, contemplation, and preparation stages; action = action and maintenance stages. ^a^ Mann–Whitney U test; ^b^ Benjamini–Hochberg (BH) false discovery rate procedure; ^c^ Cramér’s V and Rank-biserial correlation were used as effect sizes for categorical and continuous variables, respectively; ^d^ confidence intervals for Cramér’s V were obtained by inverting the noncentral chi-square distribution; ^e^ unadjusted *p*.

**Table 3:** Differences in sociodemographic characteristics, self-efficacy, and health-related factors by the combined stages of change (*N* = 208).

Variables	No-Action G ^a^ (*n* = 76)	Single-Action G ^b^ (*n* = 76)	Double-Action G ^c^ (*n* = 56)	x^2^ or H ^d^	(BH-Adjusted *p*) ^e^	Post Hoc ^f^	Effect Size ^g^	(95% CI) ^h^
	*n* (%) or M ± SD							
Age (years)	59.57 ± 6.07	60.43 ± 6.09	62.54 ± 6.42	11.82	(0.012)	a, b < c	0.06	(0.02, 1.00)
40–49	7 (9.2)	5 (6.6)	5 (8.9)	10.29	(0.035) ^i^			
50–59	25 (32.9)	24 (31.6)	6 (10.7)					
60–<70	44 (57.9)	47 (61.8)	45 (80.4)					
Sex								
Male	63 (82.9)	62 (81.6)	40 (71.4)	2.96	(0.297)		0.12	(0.00, 0.24)
Female	13 (17.1)	14 (18.4)	16 (28.6)					
Level of education								
≤High school	48 (63.2)	35 (46.1)	37 (66.1)	6.76	(0.074)		0.18	(0.00, 0.31)
≥University	28 (36.8)	41 (53.9)	19 (33.9)					
Marital status								
Married	56 (73.7)	58 (76.3)	47 (83.9)	2.02	(0.416)		0.10	(0.00, 0.22)
Single	20 (26.3)	18 (23.7)	9 (16.1)					
Employment status								
Employed	53 (69.7)	56 (73.7)	28 (50.0)	8.84	(0.031)		0.21	(0.05, 0.33)
Unemployed	23 (30.3)	20 (26.3)	28 (50.0)					
Monthly income (million won)								
<2	19 (25.0)	14 (18.4)	18 (32.1)	4.16	(0.416)		0.10	(0.00, 0.17)
2–4.9	35 (46.1)	33 (43.4)	21 (37.5)					
≥5	22 (28.9)	29 (38.2)	17 (30.4)					
Cardiovascular disease								
Yes	44 (57.9)	33 (43.4)	27 (48.2)	3.28	(0.297)		0.13	(0.00, 0.25)
No	32 (42.1)	43 (56.6)	29 (51.8)					
Self-efficacy	6.35 ± 2.08	6.88 ± 1.70	6.71 ± 2.42	3.50	(0.297)		0.02	(0.00, 1.00)
**Health-related factor**								
Healthy diet	30.13 ± 5.70	33.32 ± 5.67	34.71 ± 5.97	17.68	(<0.001)	a < b, c	0.09	(0.04, 1.00)
Self-care	65.11 ± 13.24	72.24 ± 13.30	77.68 ± 12.56	32.38	(<0.001)	a < b < c	0.16	(0.08, 1.00)
Sleep quality	6.80 ± 3.54	7.50 ± 3.86	7.23 ± 3.57	1.27	(0.531) ^i^			
<5	34 (44.7)	28 (36.8)	20 (35.7)	1.43	(0.488)		0.08	(0.00, 0.20)
≥5	42 (55.3)	48 (63.2)	36 (64.3)					
Current drinker								
Yes	43 (56.6)	33 (43.4)	25 (44.6)	3.10	(0.297)		0.12	(0.00, 0.24)
No	33 (43.4)	43 (56.6)	31 (55.4)					
Current smoker								
Yes	27 (35.5)	23 (30.3)	6 (10.7)	10.77	(0.015)		0.23	(0.07, 0.36)
No	49 (64.5)	53 (69.7)	50 (89.3)					

Note. CI = Confidence intervals; G = group; M = Mean; SD = Standard deviation. ^a^ No-action = preparation group for both physical activity and dietary management; ^b^ single-action = reclassified action group for either physical activity or dietary management; ^c^ double-action = reclassified action group for both physical activity and dietary management; ^d^ Kruskal–Wallis test; ^e^ Benjamini–Hochberg (BH) false discovery rate procedure; ^f^ Dunn’s test with Bonferroni; ^g^ Cramér’s V and Rank epsilon squared were used as effect sizes for categorical and continuous variables, respectively; ^h^ confidence intervals for Cramér’s V were obtained by inverting the noncentral chi-square distribution; ^i^ unadjusted *p*.

## Data Availability

Data available upon request due to privacy/ethical restrictions.

## Supplementary Materials

The following supporting information can be downloaded at: https://www.mdpi.com/article/10.3390/nursrep16100347/s1, Table S1. Sensitivity Analysis for Missing-Value Exclusion (*N* = 208).

## Public Involvement Statement

No public involvement in any aspect of this research.

## Guidelines and Standards Statement

This manuscript was drafted against the STROBE (Strengthening the Reporting of Observational Studies in Epidemiology) reporting guidelines for cross-sectional research.
