# Supplementary material for: Sociodemographic, Self-Efficacy, and Health-Related Factors According to the Stages of Change in Physical Activity and Dietary Management Among Patients with Metabolic Syndrome: A Secondary Analysis of a Cross-Sectional Study
Source: Nurs Rep. 2026 Sep 23;16(10):347. doi: 10.3390/nursrep16100347 (PMC13708655; doi:10.3390/nursrep16100347)
Supplement: Supplementary file 1 [file nursrep-16-00347-s001.zip › nursrep-4487841-supplementary.pdf]

Table S1. Sensitivity Analysis for Missing-Value Exclusion. (N = 208)

Variable	Classification	Raw data with missing values (N = 208)	Complete-case data (n = 203)	Imputed data (N = 208)
			n (%) or M±SD	
Age		60.68±6.25	60.72±6.17	60.68±6.25
	40−49	17 (8.2)	16 (7.9)	17 (8.2)
	50−59	55 (26.4)	54 (26.6)	55 (26.4)
	60−<70	136 (65.4)	133 (65.5)	136 (65.4)
Sex	Male	165 (79.3)	161 (79.3)	165 (79.3)
	Female	43 (20.7)	42 (20.7)	43 (20.7)
Level of education	≤ High school	120 (57.7)	118 (58.1)	120 (57.7)
	≥ University	88 (42.3)	85 (41.9)	88 (42.3)
Marital status	Married	161 (77.4)	158 (77.8)	161 (77.4)
	Single	47 (22.6)	45 (22.2)	47 (22.6)
Employment status	Employed	137 (65.9)	133 (65.5)	137 (65.9)
	Unemployed	71 (34.1)	70 (34.5)	71 (34.1)
Monthly income (million won)	< 2	51 (24.5)	48 (23.6)	51 (24.5)
	2−4.9	89 (42.8)	88 (43.3)	89 (42.8)
	≥ 5	68 (32.7)	67 (33.0)	68 (32.7)
Body mass index (kg/m^2)		26.14±3.50	26.10±3.46	26.14±3.50
	< 18.5	2 (1.0)	2 (1.0)	2 (1.0)
	18.5−22.9	34 (16.3)	33 (16.2)	34 (16.3)
	23.0−24.9	45 (21.6)	44 (21.7)	45 (21.6)
	≥ 25.0	127 (61.1)	124 (61.1)	127 (61.1)
HbA1c (%) (n = 205)		6.73±0.93	6.74±0.93	6.75±0.96
Glucose (mg/dL) (n = 203)		143.02±54.20	143.02±54.20	143.31±54.59
Waist circumference (cm) (n = 207)		92.62±8.93	92.52±8.95	92.57±8.93
Systolic blood pressure (mmHg)		124.57±15.23	124.76±15.24	124.57±15.23
Diastolic blood pressure (mmHg)		74.61±11.64	74.61±11.59	74.61±11.64
**Lipid profiles** (mg/dL)				
High density lipoprotein cholesterol (n = 204)		48.73±10.28	48.67±10.27	48.55±10.40
Low density lipoprotein cholesterol (n = 204)		60.05±28.48	60.14±28.52	59.61±28.57
Total cholesterol (n = 204)		141.46±32.54	141.56±32.59	141.45±32.45
Triglyceride (n = 204)		163.25±85.56	163.53±85.68	166.09±90.14

Cardiovascular disease	Yes	104 (50.0)	103 (50.7)	104 (50.0)
	No	104 (50.0)	100 (49.3)	104 (50.0)
Self-efficacy		6.64±2.06	6.64±2.07	6.64±2.06
**Health-related factor**				
Healthy diet		32.53±6.04	32.50±6.00	32.53±6.04
Self-care		71.10±13.96	71.10±14.0	71.10±13.96
Sleep quality		7.17±3.66	7.10±3.60	7.17±3.66
	< 5	82 (39.4)	81 (39.9)	82 (39.4)
	≥ 5	126 (60.6)	122 (60.1)	126 (60.6)
Current drinker	Yes	101 (48.6)	99 (48.8)	101 (48.6)
	No	107 (51.4)	104 (51.2)	107 (51.4)
Current smoker	Yes	56 (26.9)	55 (27.1)	56 (26.9)
	No	152 (73.1)	148 (72.9)	152 (73.1)
**Stages of behavioral change[a]**				
Physical activity	Preparation group	99 (47.6)	97 (47.8)	99 (47.6)
	Precontemplation	8 (3.9)	8 (3.9)	8 (3.9)
	Contemplation	57 (27.4)	56 (27.6)	57 (27.4)
	Preparation	34 (16.3)	33 (16.3)	34 (16.3)
	Action group	109 (52.4)	106 (52.2)	109 (52.4)
	Action	24 (11.5)	23 (11.3)	24 (11.5)
	Maintenance	85 (40.9)	83 (40.9)	85 (40.9)
Diet	Preparation group	129 (62.0)	128 (63.1)	129 (62.0)
	Precontemplation	16 (7.7)	16 (7.9)	16 (7.7)
	Contemplation	74 (35.6)	73 (36.0)	74 (35.6)
	Preparation	39 (18.7)	39 (19.2)	39 (18.7)
	Action group	79 (38.0)	75 (36.9)	79 (38.0)
	Action	21 (10.1)	19 (9.3)	21 (10.1)
	Maintenance	58 (27.9)	56 (27.6)	58 (27.9)
**Combined stages of health behavior change[b]**	No-action group	76 (36.5)	75 (37.0)	76 (36.5)
	Single-action group	76 (36.5)	75 (37.0)	76 (36.5)
	Double-action group	56 (27.0)	53 (26.1)	56 (27.0)

Note. M = Mean; SD = Standard deviation. [a] Preparation group= precontemplation, contemplation, and preparation stages; action group= action and maintenance stages. [b] No-action group =preparation group for both physical activity and dietary management; single-action group =reclassified action group for either physical activity or dietary management; double-action group =reclassified action group for both physical activity and dietary management.
